# Phenological shifts in mating and lambing timing in response to climate change in Urial wild sheep (*Ovis vignei*) populations in Iran

**DOI:** 10.1371/journal.pone.0348629

**Published:** 2026-06-04

**Authors:** Fereshteh Khaleghdadi, Farid Salmanpour, Peyman Valizadeh, Faraham Ahmadzadeh

**Affiliations:** 1 Department of Biodiversity and Ecosystem Management, Environmental Sciences Research Institute, Shahid Beheshti University, Tehran, Iran; 2 Department of Environment, Iranian Environment governmental organization, Tehran, Iran; Tshwane University of Technology, SOUTH AFRICA

## Abstract

Climate change is increasingly disrupting ecological processes across arid and mountainous biomes, with profound implications for the reproductive phenology of large herbivores. These species are especially climate-sensitive, as their breeding cycles are tightly coupled with vegetation dynamics driven by seasonal temperature and precipitation. Yet, in biodiversity-rich regions such as eastern Iran, where climate variability is acute and data are sparse, long-term phenological responses remain poorly understood. Here, we examine how reproductive timing in urial sheep (*Ovis vignei*), a mountain herbivore, responds to climatic variation across six protected areas, as climate-driven mismatches between birth timing and peak forage availability may reduce neonate survival and ultimately affect population viability and connectivity. Climate data (temperature, precipitation, snowfall, and humidity) from the nearest weather station to each study area, along with latitude and mean elevation of each habitat, were integrated using generalized linear mixed models (GLMMs) to assess phenological responses to environmental variables. Our results reveal clear regional differences in mating and lambing time. Mating time was significantly influenced by latitude, summer temperature, and autumn precipitation, with higher latitudes and autumn rainfall delaying mating, while warmer summers advanced it. In contrast, lambing timing was largely dictated by study area-level random effects, which accounted for the majority of variance, whereas fixed effects such as January temperature, snowfall, and latitude contributed only minimally, highlighting the dominant role of spatial differences among study areas in shaping lambing phenology. These findings, over the past decade, underscore the role of climate and latitude in shaping reproductive timing and highlight the urgent need to incorporate phenological data into adaptive wildlife management and habitat-specific climate resilience planning in vulnerable arid mountain ecosystems.

## 1. Introduction

The accelerating pace of climate change presents an escalating and immediate threat to global biodiversity, profoundly altering species’ physiology, morphology, phenology, and spatial distributions [1–4]. As ecosystems continue to warm and precipitation regimes shift, many species attempt to respond through behavioral, physiological, or phenological adjustments [5,6]. However, such adaptations often require multiple generations to emerge, resulting in a mismatch between the pace of environmental change and the species’ evolutionary capacity to adapt [7,8]. This lag amplifies the risk of population declines, habitat degradation, and ultimately extinction in sensitive taxa [9–11].

Yet, the impacts of climate change are neither uniform across ecosystems nor consistent among species [12–14]. Some ecosystems are exposed to more severe climatic pressures, and species differ widely in their sensitivity, plasticity, and adaptive potential [15,16]. Among the most vulnerable are large-bodied herbivorous mammals, whose survival and reproduction are closely linked to plant phenology, a trait itself tightly regulated by climatic conditions [17,18]. For these species, reproduction is largely regulated by photoperiodic cues, while warming temperatures and shifts in growing seasons may decouple the timing of resource availability from critical life-history events such as mating and birth [19–21].

Empirical studies increasingly show that migration timing in large herbivores responds to climate-driven cues, including temperature, primary productivity, and snow accumulation or melt-off, whereas reproductive phenology is primarily regulated by photoperiod, with climate effects being largely indirect [22–24]. For instance, numerous populations now migrate earlier to cooler, high-altitude summer ranges and delay their return to lowland wintering habitats [25]. Furthermore, high-elevation protected areas where livestock grazing and poaching are limited may act as critical climate refugia, buffering large mammals from adverse climate effects [26,27].

Iran, located within a global biodiversity hotspot, is experiencing disproportionate climate impacts, posing major risks to its native vertebrate fauna [28–30]. Among species of conservation concern is the urial (*Ovis vignei*), a highly seasonal breeder classified as Vulnerable (VU), which is primarily regulated by photoperiodic cues rather than direct environmental resource availability, and is additionally threatened by habitat loss and poaching [31–34]. Despite their ecological importance as seed dispersers [35] and as key prey for apex predators such as the critically endangered Asiatic cheetah (*Acinonyx jubatus venaticus*) and the endangered Persian leopard (*Panthera pardus tulliana*) [31,36,37] their reproductive and migratory phenology under climate trends remains poorly studied, particularly in Western Asia.

In this study, we explore the extent to which reproductive phenology in urial populations across eastern Iran has shifted in response to changing climatic conditions. Specifically, we aim to: (1) examine variation in the timing of mating and lambing across populations occurring along different latitudinal and elevational gradients, with the understanding that lambing timing primarily reflects mating timing given the relatively fixed gestation period in ruminants; (2) to identify which climatic variables drive these temporal changes; and (3) to evaluate long-term trends in both the timing of reproductive events and key climatic parameters. By clarifying these relationships, our findings can inform adaptive, habitat-specific management strategies to enhance species resilience, preserve functional migratory pathways, and mitigate the compounded effects of climate and anthropogenic stressors [38–41].

## 2. Materials and methods

### 2.1. Study areas

**Fig 1:**
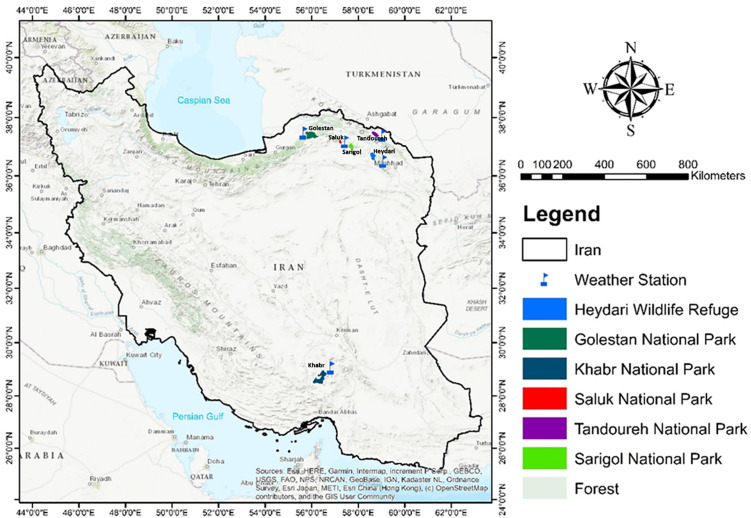
Map of the six major protected areas studied, representing the core habitats of urial in Iran, including Golestan, Saluk, Sarigol, Khabr, Tandoureh National Parks, and Heydari Wildlife Refuge. These areas span a wide range of elevations, climatic conditions, and latitudes ranging from 28.8° N in Khabr to 37.6° N in Tandoureh covering semi-humid broadleaf forests and steppes in the north to semi-arid mountainous landscapes in the south, and providing critical habitats for urial and other large mammals [40]. The map was entirely created by the authors (F. Salmanpour) using ArcGIS 10.8. Basemap and spatial data were obtained from open-source datasets compatible with the Creative Commons Attribution (CC BY 4.0) license. No proprietary or copyrighted imagery (e.g., Google Maps or Google Earth) was used‌‌.

This study was conducted across six major protected areas representing the core habitats of urial in Iran (Fig 1). These areas vary in latitude, elevation, and climate, providing a broad environmental gradient for assessing reproductive phenology. Observations took place between 2011 and 2023, indicated for each location in Table 1.

**Table 1:** Summary of mating and lambing data collection periods for each study area, along with the corresponding nearest meteorological stations used for daily synoptic weather data. Distance to the station (km) and station elevation (m) are provided for reference.

	Study area	Mating data	Lambing data	Weather Station		
				Name	Distance (km)	Elevation (m)
1	Golestan	2011-2023	2011-2023	Kalaleh Airport	~42	126
2	Heidary	2012-2022	2012-2022	Neyshabur	~48	1210
3	Saluk	2011-2020	2011-2020	Esfarayen	~35	1200
4	Sarigol	2011-2020	2011-2020	Esfarayen	~29	1200
5	Tandooreh	2016-2023	2016-2023	Dargaz	~24	508
6	Khabr	2013-2023	2013-2023	Baft	~34	2280

*Golestan National Park*: Located at 37°15′–37°35′ N in northeastern Iran, with elevations ranging from 450–2,411 m, mean temperatures of 11.5–17.5°C, and annual precipitation of 150–700 mm. Semi-humid forests and steppes provide habitat for urial and other large mammals.

*Saluk National Park:* Located at 36°38′–37°10′ N in North Khorasan, with elevations ranging from 1,150–2,170 m, mean temperature of 12°C, and annual rainfall of 280 mm. The area is characterized by semi-arid Artemisia shrublands and scattered Juniper vegetation.

*Sarigol National Park*: Located at 36°35′–37°05′ N, with elevations ranging from 1,400–2,940 m, mean temperature of 14°C, and annual rainfall of 273 mm. The landscape consists of steppe and Juniper woodlands supporting urial populations.

*Khabr National Park:* Located at 28°30′–29°00′ N in southern Iran, with elevations ranging from 1,040–3,860 m, mean temperatures of 9–21°C, and annual rainfall of 200–300 mm. The region includes mountainous semi-arid habitats with alpine to desert scrub vegetation.

*Tandoureh National Park:* Located at 37°20′–37°30′ N, with elevations ranging from 900–2,600 m, mean temperature ranging from 2.5–26°C, and annual rainfall of 370 mm. The area represents a Mediterranean to semi-arid climate with steppe and scattered Juniper vegetation.

*Heydari Wildlife Refuge:* Located at 36°35′–36°50′ N, with elevations ranging from 1,380–2,940 m, mean temperature of 12°C, and annual rainfall of 300 mm. The habitat consists of steppe and forest patches supporting significant urial populations [42].

### 2.2. Study species

The urial, the largest wild sheep species in Iran, belongs to the family Bovidae. Adult males are distinguished by long, forward-curving spiral horns and a chest mane of white fur, whereas females have shorter horns. Urials are herbivorous, diurnal, and social, inhabiting rugged hills and semi-arid mountainous landscapes with variable vegetation and limited water availability [31,43,44]. Mating occurs in mid-autumn, with a gestation period of approximately 165 days, resulting in one or two lambs born in May. Lambs are nursed for up to four months and reach sexual maturity at 1.5 years [31,43]. Adult males form small bachelor groups outside the breeding season, whereas females and young may aggregate in groups exceeding 100 individuals.

Urials are distributed across Iran, Afghanistan, Pakistan, Turkmenistan, and neighboring regions, inhabiting elevations up to 1,500–4,000 m [32,44]. They face environmental pressures from harsh climates, human disturbance, and predation by carnivores such as leopards, wolves, jackals, and foxes [31,43]. The species is currently listed as Vulnerable on the IUCN Red List, with populations declining by at least 30% over the past three generations due to illegal hunting, competition with livestock, and habitat degradation [32]. Key information on reproductive and population biology, along with habitat preferences, provides essential context for understanding phenological patterns and informing conservation strategies.

### 2.3. Data collection

Phenological monitoring of mating and lambing was conducted in long-term protected areas supporting dense urial populations, where terrain complexity and vegetation structure allowed reliable direct visual observations [42]. Surveys were primarily carried out during early morning hours, coinciding with peak activity levels and optimal visibility conditions [31,43].

Patrols were implemented in a systematic and pre-planned manner across all study areas; however, monitoring intensity increased substantially during the two critical periods of autumn mating and spring lambing when observations and data recording were conducted on a daily basis and focused on the main reproductive herds, which served as representative populations for each study habitat, allowing reliable inference of population-level reproductive timing.

All surveys were performed by Department of Environment (DOE)-trained ranger teams to minimize observer-related bias. Patrol units typically consisted of two observers and were led by experienced rangers. Monitoring followed established patrol routes that have been repeatedly used for decades within each study area. These routes varied in length among sites but were consistently surveyed over time, incorporating both motorized (vehicle and motorcycle) and pedestrian patrols. This approach reduced spatial sampling bias and ensured consistency across years and sites. To standardize observation accuracy, identical optical equipment (binoculars and field cameras) was used by all observers throughout the study areas.

Following each daily patrol, data on group size, sex composition, lamb observations during the lambing season, and male agonistic behaviors during the rut were recorded using standardized DOE data sheets by senior rangers. This protocol minimized individual recording errors and enhanced data consistency. Despite the inherent challenges of field studies in remote and rugged landscapes, the application of these standardized monitoring procedures ensured data reliability and study repeatability [45–48].

#### 2.3.1. Mating data.

Male–male fighting served as a reliable behavioral cue to determine the onset of the mating season. These behaviors were consistently observed across all six study areas, typically occurring from late October to early December. Daily patrols recorded all such events, especially male–male competitive interactions at the onset of rut leading to harem formation, using standardized observation forms. To ensure consistency, the start of the mating season was defined as the first confirmed fighting event among mature males, accompanied by sustained fighting. While occasional early bouts among subadult males were noted, these were excluded from the analysis to avoid misrepresenting population-level reproductive activity. Only collective behavioral cues from the dominant male cohort were used to establish the official onset of rutting.

#### 2.3.2. Lambing data.

During the lambing period, females often isolate themselves and conceal their neonates in dense vegetation shortly after lambing, making direct observation of births challenging. Therefore, detection of solitary females was used as an indirect indicator of hidden offspring. Observers recorded daily sightings using standardized forms provided by the Department of Environment to ensure uniform data collection. To enhance temporal accuracy, birth records were limited to herds under regular surveillance. Early outlier births of calves seen significantly before the main birthing wave were excluded to maintain temporal consistency. The onset of lambing was defined as the first observed mother–calf pair, though this likely lags actual birth by approximately 7–10 days. This conservative approach allowed for standardized interannual comparisons.

#### 2.3.3. Weather data.

Daily synoptic weather data were obtained from the nearest meteorological stations to each study area (Table 1). Four key climatic variables were extracted for analysis: mean daily temperature (°C; tm), 24-hour precipitation (mm; rrr24), the number of days with snowfall (ness), mean daily relative humidity (%; um), and maximum snow depth (mm; essmax). Data were initially recorded as daily values and then aggregated into monthly and seasonal (three-month) averages for inclusion in the models.

### 2.4. Statistical analysis

#### 2.4.1. Temporal trend analysis.

To examine temporal dynamics across the study period, linear regression models were applied with a 95% confidence interval [49]. Following the approaches of [17,21,50], we quantified both the direction and magnitude of temporal trends in reproductive events (mating and lambing) and key climatic variables. In these analyses, reproductive timing and climate parameters were treated as response variables, while year was included as the predictor variable.

#### 2.4.2. Multicollinearity check.

Prior to model construction, Variance Inflation Factor (VIF) analyses were performed to detect multicollinearity. Climatic variables with VIF values exceeding 5 were excluded to ensure model stability and interpretability [51].

#### 2.4.3. *Generalized linear mixed models (GLMMs).*

To assess how climate influences mating and lambing phenology in wild urial populations, separate generalized linear mixed models (GLMMs) were developed [52]. This approach allowed the detection of phase-specific climate effects on reproductive timing, with study area included as a random effect and climatic variables, mean elevation, and latitude as fixed auxiliary predictors. Each model incorporated between one and three predictor variables to avoid overfitting.

##### 2.4.3.1. *Testing model assumptions:*

Candidate models combining non-collinear predictors were ranked using the corrected Akaike Information Criterion (AICc), which includes a correction for small sample sizes, and associated model weights to balance explanatory power with model simplicity. Models with ΔAICc ≤ 2 were considered top-ranked candidates, following the framework of [53].

##### 2.4.3.2. *Model validation:*

All candidate GLMMs were evaluated using standard diagnostic procedures to ensure model reliability and predictive accuracy [54]. Model validation included assessment of overdispersion, zero-inflation, and residual uniformity. Marginal and conditional R² values were calculated to quantify the proportion of variance explained by fixed effects alone and by both fixed and random effects, respectively. These diagnostics confirmed that the selected models were robust, free from major biases, and suitable for inference on reproductive phenology in urial populations.

##### 2.4.3.3. *Interpretation of results:*

Final parameter estimates, including standard errors, β coefficients, t-statistics, and p-values, were extracted using the tidy function from the broom package, facilitating interpretation of climatic influences on urial reproductive timing.

All analyses were conducted in R version 4.4.3 [55] using base functions and additional packages (car, DHARMa, broom, MuMIn) for model fitting, diagnostic evaluation, and model selection.

Declarations

- Authors’ contributions

Fereshteh Khaleghdadi: Writing - original draft; Writing - review & editing; Filed work; Methodology; Data collection

Farid Salmanpour: Writing - original draft; Writing - review & editing; Project administration; Formal analysis; Software; Methodology; Data collection

Peyman Valizadeh: Writing - review & editing; Data collection

Faraham Ahmadzadeh: Writing - review & editing; Supervision;

- Ethics approval and consent to participate

No specific permits were required for this study. All data were collected under the supervision and collaboration of Mr. Peyman Valizadeh, an official employee of the Department of Environment, Iranian Environment governmental organization, in accordance with local regulations and standard ethical practices.

Consent for publication

Not applicable

- Availability of data and materials

Datasets analyzed during the current study are available on Figshare as https://figshare.com/s/f156696520eac98de8a3

Competing interests

The authors confirm that they have no conflicts of interest with respect to the work described in this manuscript.

- Funding

This research did not receive any specific grant from funding agencies in the public, commercial, or not-for-profit sectors

- Authors’ information

Not applicable

- Footnotes

Not applicable

## 3. Results

**Fig 2:**
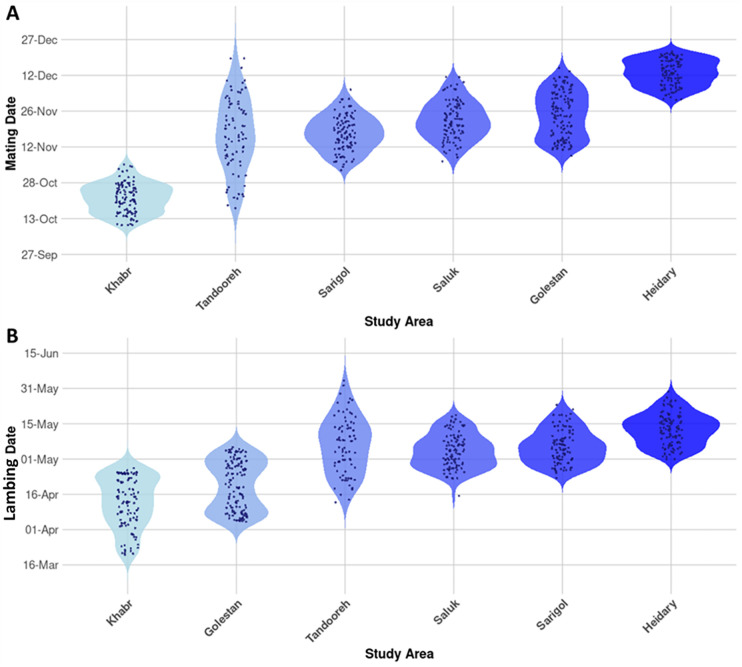
Spatial variation in reproductive phenology across the studied habitats. (A) Mating onset differed among regions, with Khabr showing the earliest activity (10–28 October) and Heidary the latest (early–late December); other areas exhibited intermediate timing. (B) mating showed a parallel pattern, occurring earliest in Khabr (late March–late April), followed by Golestan (early April–early May), with intermediate timing in Saluk and Sarigol, and the latest births in Heidary (early–late May)‌‌.

Our findings showed that the onset of mating varied among the studied habitats (Fig 2A). Khabr consistently exhibited the earliest activity, occurring between 10 and 28 October. Tandooreh followed, displaying a more extended period of mating events from late October to late December. In Golestan, the mating season spanned from 11 November to 15 December, whereas Sarigol and Saluk showed intermediate timing, generally from early November to early December. Heidary recorded the latest mating activity, ranging from early to late December.

Mirroring the observed variation in mating, the timing of lambing also differed markedly among the regions (Fig 2B). Khabr exhibited the earliest births, with mating commencing as early as late March and extending into late April, indicating the earliest reproductive onset among all studied areas. Golestan followed, with births beginning in early April (from 4 April) and continuing until early May, with a clear concentration during mid- to late April. Saluk and Sarigol showed intermediate timing, with most births occurring from late April to mid-May. Tandooreh displayed a broader and later window, spanning from mid-April to late May. Heidary consistently recorded the latest mating, extending from early to late May.

**Fig 3:**
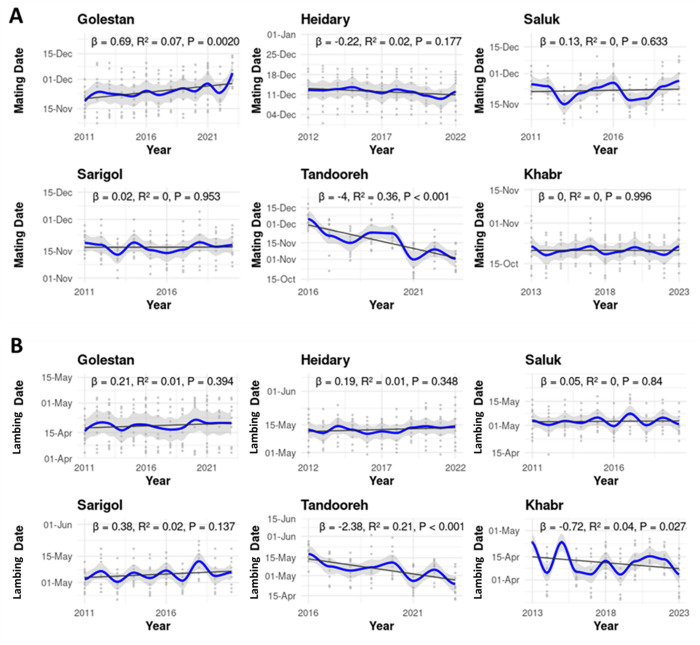
Interannual trends in mating (A) and lambing (B) dates across study regions, showing strong year-to-year variability and a clear advancing trend only in Tandooreh.

Across regions, both mating and lambing dates exhibited noticeable interannual variability, with no consistent long-term directional trend in most areas (Fig 3). Temporal fluctuations were evident in all populations, indicating year-to-year shifts in reproductive timing rather than monotonic change. In contrast, Tandooreh showed a distinct pattern, with a significant advancing trend toward earlier dates in both mating and lambing over the study period.

The top‐ranked mixed‐effects model explaining variation in mating timing included latitude, mean summer temperature, and autumn precipitation as fixed effects, with study area treated as a random intercept (Supplementary 1). This model received the strongest support among candidate models (ΔAICc = 0, weight = 0.194), indicating a meaningful contribution of both spatial and climatic drivers. Mating date increased significantly with latitude (β = 4.24 ± 1.46, t = 2.91, p = 0.044), suggesting progressively later mating at higher latitudes. In contrast, higher mean summer temperatures lead to earlier mating times (β = −3.37 ± 1.13, t = −2.98, p = 0.004), whereas increased autumn precipitation delayed mating (β = 5.04 ± 2.46, t = 2.05, p = 0.045). Notably, no competing models had ΔAICc < 2, highlighting the clear superiority of this model (Table 2).

**Table 2:** Summary of top-ranked models for mating and lambing timing. For each model, we report model selection metrics including AICc, log-likelihood (logLik), ΔAICc, and model weight, as well as coefficients (± SE), t-values, and p-values for all predictor variables. Mating timing was significantly influenced by latitude, summer temperature, and autumn rainfall, while lambing timing was primarily affected by January temperature, with snowfall showing no significant effect.

Index	Rank	AICc	logLik	∆AICc	Weight	Variable	Coefficient ± SE	t	p
**Mating**	Model 1	399.787	−193.143	0	0.194	Intercept	249.827 ± 55.93	4.47	0.006
						Latitude °	4.243 ± 1.456	2.91	0.044
						Temperature (°C)- Summer	−3.368 ± 1.129	−2.98	0.004
						Rainfall (mm) – Autumn	5.043 ± 2.461	2.05	0.045
**Lambing**	Model 1	385.825	−186.163	0	0.101	Intercept	103.19 ± 6.675	15.46	<0.001
						Temperature (°C) – January	1.163 ± 0.411	2.83	0.006
						Number of snowfall reports (mm) – January	2.884 ± 5.471	0.53	0.6
						Number of snowfall reports (mm) – March	5.186 ± 20.676	0.25	0.803
	Model 2	386.286	−186.393	0.461	0.08	Intercept	9.142 ± 64.837	0.14	0.895
						Latitude °	2.643 ± 1.803	1.47	0.219
						Temperature (°C) – January	1.064 ± 0.378	2.82	0.007
						Number of snowfall reports (mm) – March	10.873 ± 17.023	0.64	0.973
	Model 3	387.784	−187.142	1.959	0.038	Intercept	15.269 ± 53.117	0.29	0.788
						Latitude °	2.617 ± 1.478	1.77	1.77
						Number of snowfall reports (mm) – January	−2.83 ± 5.433	−0.52	0.605
						Number of snowfall reports (mm) – March	0.752 ± 22.091	0.03	0.973

For mating timing, the top-ranked model (*Latitude + Summer temperature + Autumn rainfall*, ΔAICc = 0) demonstrated excellent predictive performance. Diagnostics indicated no overdispersion (dispersion p = 0.884) and no zero-inflation (p = 1), while residual uniformity was adequate (p = 0.096). The model explained a substantial proportion of variance, with marginal R² = 0.511 and conditional R² = 0.918, highlighting the strong contribution of fixed effects and the added explanatory power of study area-level random effects (Random Var = 119.82). These results suggest that spatial location and seasonal climatic variables are reliable predictors of mating phenology, and the model can effectively account for variation among different study areas.

The top-ranked models indicated that lambing timing was primarily influenced by January temperature, with higher temperatures associated with later lambing (Model 1: β = 1.16 ± 0.41, t = 2.83, p = 0.006; Model 2: β = 1.06 ± 0.38, t = 2.82, p = 0.007). Snowfall in January and March, as well as latitude, showed no significant effects on lambing timing across the candidate models (Table 2).

For lambing timing, three models fell within the ΔAICc ≤ 2 threshold, all showing robust performance. All models exhibited adequate dispersion (p = 0.742–0.934) and no zero-inflation (p = 1), with residual uniformity p-values ranging from 0.046 to 0.273. Conditional R² values were high (0.900–0.935), indicating that study area random effects captured most of the variance, whereas marginal R² varied more widely (0.035–0.322), reflecting the smaller relative contribution of fixed effects.

Among the climatic variables identified by the models as influential, mean summer temperature, autumn rainfall, and January snowfall exhibited significant interannual variability. However, no meaningful long-term trends were detected across any of the studied areas during the study period (p > 0.05). This indicates that despite short-term fluctuations, these key climatic factors have remained relatively stable regionally, without any discernible directional changes that could affect local ecological patterns.

## 4. Discussion

Our study revealed clear spatial variation in reproductive phenology across habitats. Mating onset occurred earliest in Khabr and latest in Heidary, with other areas showing intermediate timing. lambing followed a parallel pattern, with earlier mating generally leading to earlier births, despite substantial interannual variability. Overall, reproductive timing fluctuated from year to year, with no consistent long-term trends in most regions, except for Tandooreh, which showed a significant advancement toward earlier mating and lambing. Latitude and seasonal climatic variables, including summer temperature and autumn rainfall, were key predictors of mating phenology, while January temperature primarily influenced lambing timing.

Our results highlight the important role of latitude in shaping the timing of reproductive events in large herbivores. Populations at lower latitudes showed earlier reproductive phenology compared to those at higher latitudes. Specifically, the urial population in Khabr National Park, the southernmost study site (28°30′–29°00′ N), mated 10–20 days and gave birth 5–15 days earlier than the average of five other populations located between 36°–37°50′ N. These patterns align with previous studies showing that northern populations of Artiodactyla tend to exhibit delayed reproductive phenology compared to southern populations [56–60]. Our results also showed that, on a per-degree latitude basis, mating occurred approximately 1.2–2.7 days earlier, and lambing approximately 0.6–2 days earlier for each degree decrease in latitude, which is consistent with the findings of [56] for red deer in France and Norway, where mating was delayed by 2.5–3 days and lambing by 1.5–2 days for each degree increase in northern latitude.

Despite similar latitudes, five study populations showed differences in mating and lambing timing, with mating varying by 30–45 days. Tandooreh mated in late October to early November, Golestan, Saluk, and Sarigol from early November to mid-December, and Heydari Wildlife Refuge from early to late December. Lambing in Golestan occurred about 30 days earlier than in Heydari. Although elevation was not statistically significant in our analyses, it may indirectly influence reproductive timing through its effects on local climate. This is consistent with Hopkins’ Bioclimatic Law [61], which predicts that a 1° increase in latitude has a phenological effect similar to an approximately 120 m increase in elevation under equal conditions. Such an indirect effect of elevation aligns with previous studies on mouflon (*Ovis gmelini*) in western Iran [62], and red deer (*Cervus elaphus maral*) in northern Iran [63], as well as other ungulates, which show delayed reproductive phenology in populations inhabiting higher elevations [5,18,60,64]. Mountain-dwelling Artiodactyla also exhibit habitat-dependent variation in mating patterns [65]. Despite variations among the studied populations, our findings consistent with previous studies [62,63] indicate that in habitats where mating occurs earlier, lambs are also born significantly earlier. Furthermore, our results suggest that urial populations tend to mate earlier during summers that are initially warmer and subsequently drier. These climatic conditions likely enhance the growth and availability of high-quality forage, improving nutritional status and enabling earlier reproduction. This pattern is consistent with observations in mouflon in western Iran [62] and red deer in northern Iran [63], as well as with broader evidence that reproductive phenology in Artiodactyla, even when determined by photoperiod, is also additionally influenced by climate via its effects on food resources [17,19,20,50,66–68]. Higher ambient temperatures during gestation also tend to advance birth dates, likely by providing earlier access to nutritious forage during critical reproductive stages [69–71].

Previous studies have demonstrated that increasing temperatures can disrupt key reproductive events, such as estrus and parturition, potentially decoupling them from periods of peak food availability [2,18–20,67,68,72–74]. Such phenological mismatches may reduce offspring survival particularly among male juveniles alter population sex ratios, and ultimately compromise long-term population persistence and resilience. Climate change has also been shown to disproportionately affect large mammals at lower latitudes and elevations, often promoting range shifts toward higher elevations or latitudes as temperatures rise [25,58,75,76]. In contrast to these broader patterns, our results indicated that, except for the population in Tandooreh National Park, no significant changes were detected in the timing of mating or lambing across the other study populations, nor were clear long-term trends observed in the examined climatic variables. Nevertheless, in Iran, the adverse impacts of climate change on biodiversity particularly within habitats supporting large Artiodactyla are becoming increasingly evident [29,77,78], while urial populations are simultaneously exposed to additional pressures such as poaching and habitat loss [31,32].

In this study, we focused on six study areas examining mating and lambing periods within core urial populations, without accounting for variation in population size, age–sex structure, or populations occurring outside protected areas or across different habitat types. Consequently, the magnitude and direction of phenological responses to climatic variability may differ among systems depending on ecological context, including habitat extent, population density, climatic heterogeneity, protection status, and landscape fragmentation [75–77]. Future work should therefore incorporate population structure, demographic processes, and vegetation dynamics across a broader range of habitats to improve ecological inference and conservation relevance.

Furthermore, we explicitly acknowledge that lambing timing is ultimately constrained by mating timing, while gestation length in large-bodied endotherms such as urial is expected to remain relatively stable. However, the mating-to-lambing interval used here is a population-level, behavior-based proxy rather than an exact measure of physiological gestation, as mating onset was defined by sustained rutting behavior and lambing onset by first observed mother–offspring pairs, which likely lag actual birth events by several days. All analyses were conducted at the population and annual mean level, and no inference regarding individual-level mating-to-birth linkage is intended; accordingly, the results should be interpreted as population-level phenological patterns rather than precise individual reproductive trajectories.

## Supporting information

S1 File Supplementary: Detailed phenological data for Urial Wild Sheep (*Ovis vignei*) Populations. (DOCX)

S2 File Supplementary. (XLSX)
