# Supplementary material for: Phenological shifts in mating and lambing timing in response to climate change in Urial wild sheep (Ovis vignei) populations in Iran
Source: PLoS One. 2026 Jun 4;21(6):e0348629. doi: 10.1371/journal.pone.0348629 (PMC13235904; doi:10.1371/journal.pone.0348629)
Supplement: S1 File — (DOCX) [file pone.0348629.s001.docx]

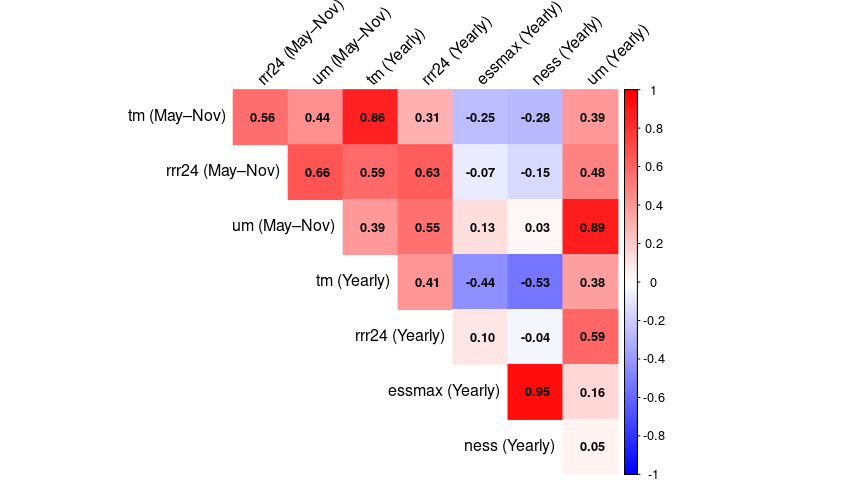


| Variance Inflation Factor (VIF) | |
| --- | --- |
| Assessment of multicollinearity among predictors in the linear model | |
| Variable | VIF |
| tm | 3.19 |
| rrr24 | 3.53 |
| um | 5.29 |
| rrr24Y | 2.93 |
| essmaxY | 1.18 |

| Comparison of Linear Models for Mating Phenology | | | | | | | |
| --- | --- | --- | --- | --- | --- | --- | --- |
| Based on AICc, BIC, logLik, and Akaike Weight | | | | | | | |
| Model | AICc | BIC | logLik | df | formula | Delta_AICc | Weight |
| **model4** | **541.38** | **547.40** | **−267.49** | **2** | **JulianDay ~ essmaxY** | **0.00** | **0.39** |
| model1 | 543.33 | 549.35 | −268.46 | 2 | JulianDay ~ tm | 1.95 | 0.15 |
| model5 | 543.45 | 551.34 | −267.38 | 3 | JulianDay ~ tm + rrr24 | 2.07 | 0.14 |
| model2 | 543.53 | 549.56 | −268.56 | 2 | JulianDay ~ rrr24 | 2.15 | 0.13 |
| model3 | 543.62 | 549.64 | −268.61 | 2 | JulianDay ~ rrr24Y | 2.24 | 0.13 |
| model6 | 545.69 | 555.36 | −267.32 | 4 | JulianDay ~ tm + rrr24 + rrr24Y | 4.31 | 0.04 |
| model7 | 546.86 | 558.22 | −266.68 | 5 | JulianDay ~ tm + rrr24 + rrr24Y + essmaxY | 5.48 | 0.03 |

| Linear Model Summary (Model 1) | | | | |
| --- | --- | --- | --- | --- |
| Estimates, Standard Errors, Z-values, and P-values | | | | |
| Variable | Estimate | Std_Error | Z_value | P_value |
| **(Intercept)** | **311.523** | **2.406** | **129.467** | **0.000** |
| essmaxY | 27.277 | 17.587 | 1.551 | 0.126 |
| Dispersion ratio: NA | | | | |

testDispersion(res)

DHARMa nonparametric dispersion test via sd of residuals fitted vs. simulated

data: simulationOutput

dispersion = 0.98327, p-value = 0.992

alternative hypothesis: two.sided

> testZeroInflation(res)

DHARMa zero-inflation test via comparison to expected zeros with simulation under H0 = fitted model

data: simulationOutput

ratioObsSim = NaN, p-value = 1

alternative hypothesis: two.sided

> testZeroInflation(res)

DHARMa zero-inflation test via comparison to expected zeros with simulation under H0 = fitted model

data: simulationOutput

ratioObsSim = NaN, p-value = 1

alternative hypothesis: two.sided

> testUniformity(res)

Asymptotic one-sample Kolmogorov-Smirnov test

data: simulationOutput$scaledResiduals

D = 0.13016, p-value = 0.2362

alternative hypothesis: two-sided


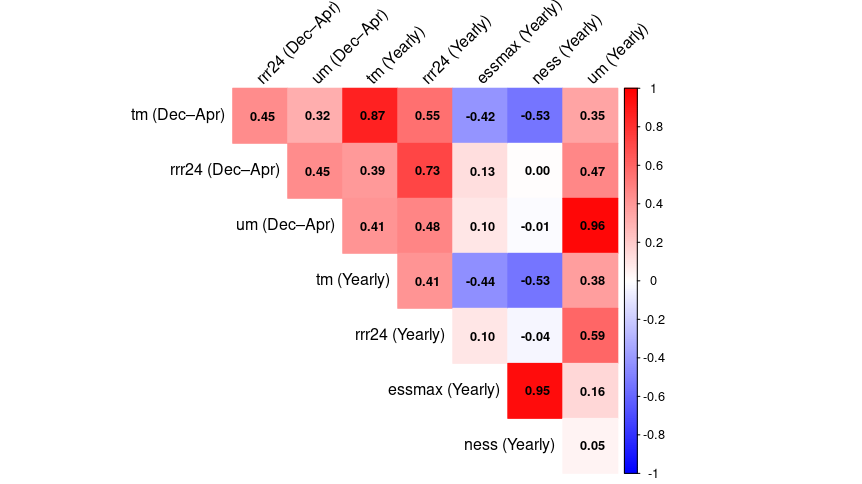


| Variance Inflation Factor (VIF) | |
| --- | --- |
| Assessment of multicollinearity among predictors in the linear model | |
| Variable | VIF |
| tm | 2.11 |
| rrr24 | 3.80 |
| um | 1.37 |
| rrr24Y | 5.31 |
| essmaxY | 1.19 |

| Comparison of Linear Models for Mating Phenology | | | | | | | |
| --- | --- | --- | --- | --- | --- | --- | --- |
| Based on AICc, BIC, logLik, and Akaike Weight | | | | | | | |
| Model | AICc | BIC | logLik | df | formula | Delta_AICc | Weight |
| **model6** | **469.78** | **479.45** | **−229.37** | **4** | **JulianDay ~ tm + rrr24 + um** | **0.00** | **0.60** |
| model7 | 470.60 | 481.96 | −228.55 | 5 | JulianDay ~ tm + rrr24 + um + essmaxY | 0.82 | 0.40 |
| model1 | 487.71 | 493.73 | −240.65 | 2 | JulianDay ~ tm | 17.93 | 0.00 |
| model5 | 488.41 | 496.29 | −239.86 | 3 | JulianDay ~ tm + rrr24 | 18.63 | 0.00 |
| model2 | 500.08 | 506.10 | −246.84 | 2 | JulianDay ~ rrr24 | 30.30 | 0.00 |
| model3 | 510.71 | 516.73 | −252.15 | 2 | JulianDay ~ um | 40.93 | 0.00 |
| model4 | 512.22 | 518.25 | −252.91 | 2 | JulianDay ~ essmaxY | 42.44 | 0.00 |

| Linear Model Summary (Model 1) | | | | |
| --- | --- | --- | --- | --- |
| Estimates, Standard Errors, Z-values, and P-values | | | | |
| Variable | Estimate | Std_Error | Z_value | P_value |
| **(Intercept)** | **103.180** | **7.037** | **14.662** | **0.000** |
| **tm** | **−3.188** | **0.657** | **−4.855** | **0.000** |
| **rrr24** | **−5.211** | **1.720** | **−3.029** | **0.004** |
| **um** | **0.548** | **0.113** | **4.829** | **0.000** |
| Dispersion ratio: NA | | | | |

> testDispersion(res)

DHARMa nonparametric dispersion test via sd of residuals fitted vs. simulated

data: simulationOutput

dispersion = 0.95104, p-value = 0.784

alternative hypothesis: two.sided

> testZeroInflation(res)

DHARMa zero-inflation test via comparison to expected zeros with simulation under H0 = fitted model

data: simulationOutput

ratioObsSim = NaN, p-value = 1

alternative hypothesis: two.sided

> testUniformity(res)

Asymptotic one-sample Kolmogorov-Smirnov test

data: simulationOutput$scaledResiduals

D = 0.050921, p-value = 0.9967

alternative hypothesis: two-sided
